# Generative Models as an Emerging Paradigm in the Chemical Sciences

**DOI:** 10.1021/jacs.2c13467

**Published:** 2023-04-13

**Authors:** Dylan M. Anstine, Olexandr Isayev

**Affiliations:** † Department of Chemistry, Mellon College of Science, Carnegie Mellon University, Pittsburgh, Pennsylvania 15213, United States

## Abstract

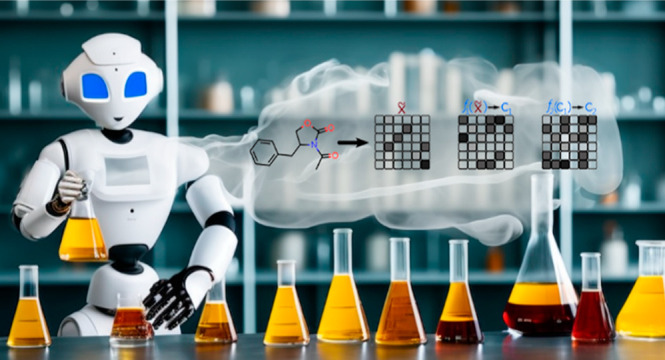

Traditional computational approaches to design chemical species are limited by the need to compute properties for a vast number of candidates, e.g., by discriminative modeling. Therefore, inverse design methods aim to start from the desired property and optimize a corresponding chemical structure. From a machine learning viewpoint, the inverse design problem can be addressed through so-called generative modeling. Mathematically, discriminative models are defined by learning the probability distribution function of properties given the molecular or material structure. In contrast, a generative model seeks to exploit the joint probability of a chemical species with target characteristics. The overarching idea of generative modeling is to implement a system that produces novel compounds that are expected to have a desired set of chemical features, effectively sidestepping issues found in the forward design process. In this contribution, we overview and critically analyze popular generative algorithms like generative adversarial networks, variational autoencoders, flow, and diffusion models. We highlight key differences between each of the models, provide insights into recent success stories, and discuss outstanding challenges for realizing generative modeling discovered solutions in chemical applications.

## Introduction

For many researchers, it is natural to frame the chemical or material discovery process in the context of the scientific method. Research practices can be contorted to fit a cyclic workflow that consists of problem identification, proposal of a testable hypothesis, computational calculations or experimental measurement, data analysis, and refinement/revisitation of the original lack of knowledge.^1^ The merit of the scientific method is irrefutable; however, it oftentimes leads to a so-called Edisonian approach to research, where systematic improvement occurs by means of human-directed trial-and-error experimentation. Several key points should be highlighted. Most commonly touted, Edisonian research lacks the efficiency required to solve complex challenges with large chemical spaces.^2−4^ This is a deficit by design. The traditional scientific method always keeps the end molecule, material, or property in mind, but characterization does not occur until several steps into the workflow. Therefore, time-consuming development and application of synthetic techniques, sample preparation, and/or modeling practices are required before judging the quality of novel materials and molecules.^5^ The nature of the discovery task being pursued is also a point of question. The forward scientific method performs best when systematic improvement can be readily achieved by heuristics and the collection of data available. However, high-performance materials and exemplary molecules may not and, for many problems, will not resemble existing species.^6^ These cases require substantial innovation beyond current knowledge to achieve the target aim.^7,8^ A paradigm of inverse design has emerged to avoid these limitations, where the target is it at the forefront of the discovery process and candidates are generated down workflow.^9,10^

Supervised learning systems can be broadly categorized into two types of models depending on their function: discriminative or generative. To describe these model types in the context of the chemical sciences, we will refer to chemical species as inputs (x) and properties or functionality as targets (y). A discriminative model is defined by learning targets conditioned on inputs: p(y|x). As an example, one might consider training a simple model to infer solubility (y) from chemical topology (x_1_) and molecular weight (x_2_). Discriminative models are commonly applied to screen known or related compounds, where rapid assessment is needed to down select from a large pool of candidates. These processes require insight from human chemists to gather potential candidates, which, depending on the design of experiment, enforces limitations on the novelty of chemical compounds. In contrast, a generative model exploits the joint probability of an input with a target: p(x,y).^4^ The overarching idea of generative modeling is to implement a system that produces novel molecules or materials that are likely to have designated {y}. Effective inverse design relies on biasing chemical species production toward the target(s), where generative models are one strategy receiving substantial interest.^11^ Conceptually, the novelty of generated compounds is bound by the production mechanism and not the human chemist. There is no requirement to amass a data set of candidates based on hypothesized chemical mechanisms because it is the role of the generative model to produce such species using an abstract high-dimensional representation. This gives rise to the idea of machine-driven hypotheses that can augment or, in some cases, replace traditional hypothesis formulation.^12^ Leveraging generative modeling as a vehicle for abstract hypothesis generation is an emerging strategy that we anticipate will be crucial for overcoming challenges across the chemical sciences.

One factor driving the realization of generative models is the widespread adoption of machine learning and data-driven research.^11,13−16^ This has been bolstered by continuous advances in accelerated computational power, for example, the advent of exascale computing.^17,18^ There is also a well-developed software ecosystem of ML tools, support for automatic differentiation, and collection of well-documented tutorials. This leads to a modest barrier to entry for those looking to pursue data-driven chemical research, such as the development of generative models for molecular discovery.

From our viewpoint, construction of effective generative models is among the greatest opportunities available in modern chemical research. They have a conceivable ability to drive autonomous scientific discovery and, as a result, can lead to a reallocation of human scientific creativity.^19−22^ Generative models can also act as key components of emerging robotic discovery platforms. They have a perceived ability to accelerate the rate at which we find solutions to pressing issues: a feat that can be achieved by acting on complex chemical and material underpinnings that could be difficult to understand with low-dimensional thinking. The goal of this perspective is not to address the particulars of implementing generative models. Although, it is worth commenting that the difficulties of developing and applying generative models should not go underappreciated. Instead, we aim to give an overview of actively explored tools and place generative modeling challenges faced across the chemical sciences into proper context. In each section we highlight seminal works and select reports that use innovative strategies, which provide direction for those looking to investigate generative modeling. Despite the field of generative models being in a relatively early stage, results reported up to this point have shown promise.^23−26^ To support breakthroughs in generative models, the second half of this perspective focuses on topics that will need to be addressed for future developments.

### Generative Models

Generative modeling methods are numerous with diverse inner workings.^4^ Despite this diversity, the ultimate aim is shared: explore unknown regions of chemical design space to find high-performing molecules or materials that can be readily synthesized and applied. Considering the generative models reported thus far, computational chemists have mainly achieved the first few words of this aim. Many studies report the identification of high-performing species, yet those following through with experimental validation are scarce. Exceptions exist, for example, the recent work of Korshunova et al.^27^ or the landmark report by Zhavoronkov et al.,^28^ and these successes support that generative models can directly address chemical science challenges. Including experimental validation is crucial to avoid generative modeling for the sake of generative modeling. Commonalities across successful generative model-to-experimental applications are multigroup multi-institution collaborations, which we strongly encourage, and the pursuit of an end-to-end research design. In our experience, modern synthetic chemists and chemical engineers are eager to use generative modeling results, especially if synthetic outlines can be provided, but the models tend to fall short.^29^ Thus, to assist in realizing future discoveries that start *in silico sine populo*, we first expand upon key generative modeling methods and concepts that are being explored by computational chemists. It is worth emphasizing that a consensus on the best generative strategy does not exist, and as a result, we make a point to highlight important advantages and disadvantages between approaches to guide model selection.

### Training with Reinforcement Learning

A high-level definition of reinforcement learning (RL) is that it is a framework to describe the process of improving ability through interactions between states, actions, and rewards.^30^ In the context of molecule or material discovery, actions can be thought of as selecting functional motifs, sequential building of chemical structures, or autoregressive species construction, to name a few. The selection of molecule or material building actions based on the current state of the system is referred to as the policy. We are cautious in providing a single encompassing definition for states and rewards because their form is often diverse depending on the demands of the discovery task. Instead, we demonstrate their role using a simple example where SMILES strings of molecules are generated autoregressively. In this case, the state of the RL system describes the ordering and characters composing the generated SMILES string, which is updated after every action. Following the generation of a complete SMILES string, a reward is given in accordance with the perceived value the molecule possesses for the target application. In policy gradient methods, i.e., those aimed at refining action selection, the reward signal determines the model parameter updates and, therefore, is responsible for guiding future actions.^31^ Most RL frameworks operate with the goal of maximizing the cumulative sum of rewards. For generative models aimed at discovery, an RL system seeks to leverage accumulated experience of testing molecules and materials for a target purpose to build better species with each training cycle.^26^

**Figure 1:**
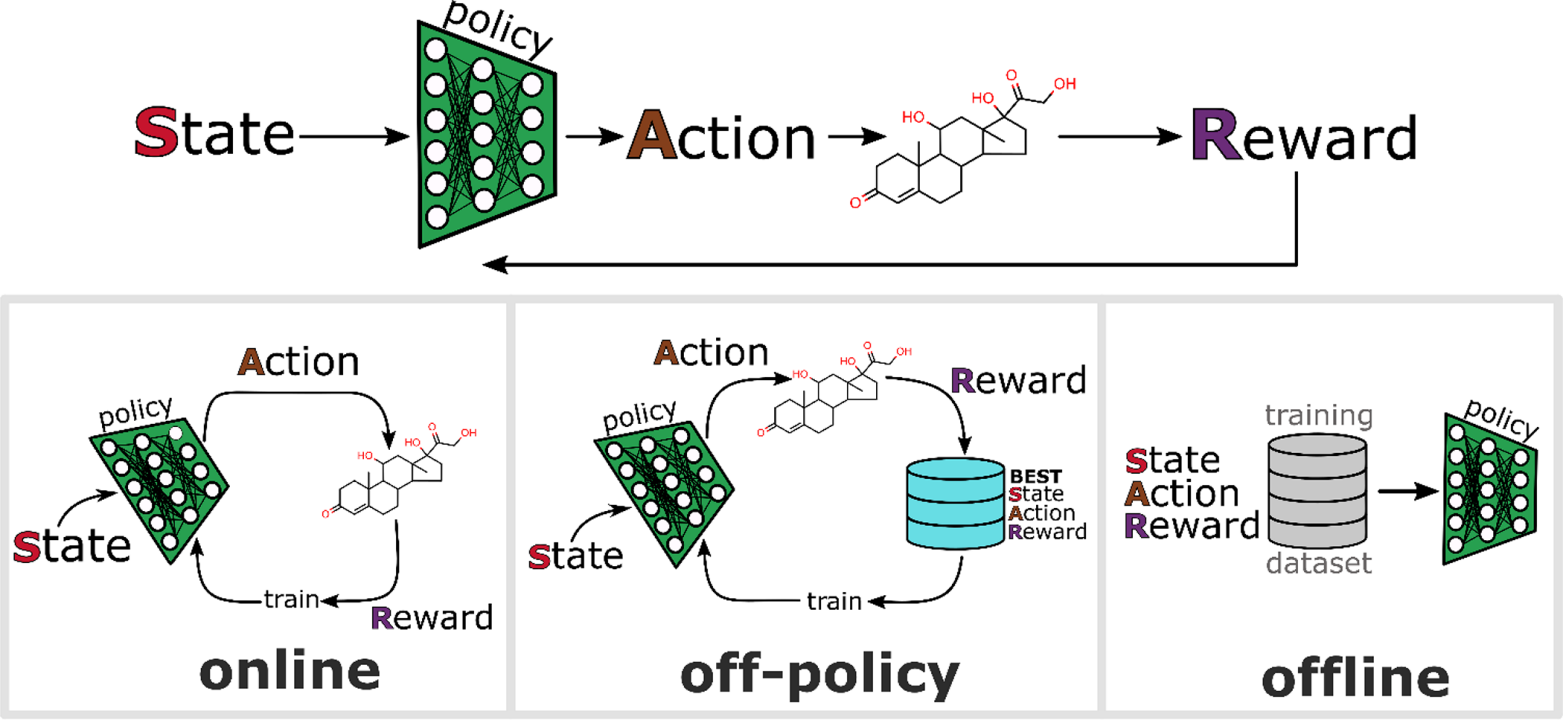
Illustration of reinforcement learning (RL) as an approach to generate chemical species. Training strategies include online, off-policy, and offline. In online RL, the most up-to-date policy is used to generate molecules. Off-policy RL carries out policy updates using a stored collection of best states, actions, and rewards. In offline RL, the training data set is external, and the policy optimization is akin to a traditional supervised learning problem.

Training an RL generative model is carried out with one of three strategies: online, off-policy, and offline (see Figure 1).^32^ The distinguishing feature between these strategies is the role of the species discovery policy. In online RL, model training occurs using states, actions, and rewards that are accumulated using the most up-to-date policy. In other words, the policy updates occur using only experiences that are produced by acting upon its current understanding of high-performing molecules or materials. Off-policy RL is a modification of the online training approach that supplements current experiences with those from prior policies. This strategy is useful because it provides a mechanism to leverage previously generated high-performing chemical species that may have moved outside the distribution generated under the current policy. It is worth commenting that an over-reliance on past chemical species can inhibit exploration. Offline RL is defined by fitting a policy to a previously accumulated data set; i.e., the experiences used to train the model are independent. Training a generative model using a predetermined collection of data is akin to standard supervised learning strategies and can be effective at mitigating the training difficulties faced by online and off-policy RL.

There is a trade-off between RL training strategies. An online generative model can traverse large distances in chemical design space. However, its efficiency, ability to avoid local minima, and ease of training are less than that of offline RL. In contrast, offline RL can become reliant on the initial data set, and therefore, its ability to discover *de novo* molecules and materials may be limited. Off-policy methods occupy a midpoint on these trade-offs. By our assessment, online and off-policy training approaches are favored by the molecule and material generative models reported thus far, but we highlight that this should not be equated to superiority over offline training.

It is common for property-guided RL generative models to be developed within the framework of so-called actor-critic methods.^33^ The actor is a neural network that carries out the policy to suggest molecules or materials, and the critic is a discriminator that judges the value of the generated chemical species and/or actions taken. One of the earliest reports of using a policy optimization method for *de novo* molecule generation was in the ReLeaSE strategy of Popova et al.^26^ The authors trained a recurrent neural network to generate SMILES strings and a discriminator that infers molecular properties from the generator output. With each discovery cycle, the model learned to suggest SMILES strings that maximize rewards administered proportionally to the target property. The DeepFMPO model of Ståhl et al. uses temporal difference learning to train a molecule generator to modify molecular fragments using a precompiled library of structures.^34^ Their method has demonstrated potential for lead optimization through exploring combinatoric chemical spaces, but the extent that exploratory ability is limited by the fragment library is unclear. A report by Gottipati et al. utilized a deep deterministic policy gradient approach to explore synthetically accessible regions of chemical space.^35^ The authors used RL to select combinations of reactants and a reaction template to form optimal product molecules. Regarding software availability, open-source RL platforms for molecular discovery are also emerging; for example, see REINVENT 2.0.^36^ The reports described above are a select sampling of RL generative models that highlight their growing prevalence in molecular discovery.

One of the significant benefits of RL-based generative models is that an existing data set of molecules or materials is not strictly required. Assuming the reward signal is well-defined and the state-action space is mappable, policy gradient methods can explore molecules and materials by learning to construct them. Our discussion of RL-based generative models has primarily focused on actor-critic methods for policy optimization. However, other approaches such as value function learning have been applied to generate molecular species; see Zhou et al.^37^ A range of new concepts from computer science are also being integrated into molecular generative models; for example, Thiede et al. recently applied the concept of curiosity from the field of intrinsic reward formulations.^38^

RL is a rapidly developing area of research with new methodologies constantly emerging. We envision that a strong connection between the chemical sciences and the evolving field of RL will lead to many future scientific breakthroughs.

### Generative Adversarial Networks

The fundamental components of a generative adversarial network (GAN) are two independent neural networks, which are termed the generator and discriminator.^39^ These networks are trained in opposition: the generator’s purpose is to suggest output that is deceiving to the discriminator, i.e., bares resemblance to a certain distribution of data. This framework can be conceptually straightforward to understand by using an example of molecule generation. The generator is trained to produce inputs, i.e., molecules, for the discriminator to judge, and model parameters are updated to maximize the number of mischaracterizations performed by the discriminator. An initial training data set of molecules and noisy inputs is used to train a discriminator whose role is to classify the data as corresponding to a real molecule or a generated species. The aim is that, given enough data, the discriminator develops an abstract chemical understanding that allows it to reasonably classify the realism of molecules. Training of these networks occurs in an iterative back-and-forth fashion, where each network is expected to perform better at its task with each training cycle. The point where improvement is no longer possible for either network without changing the other is referred to as Nash Equilibrium.^40^ The generator can then be utilized as a standalone generative model because it has learned to suggest realistic molecular species. This example illustrates the overarching concepts of GANs.

**Figure 2:**
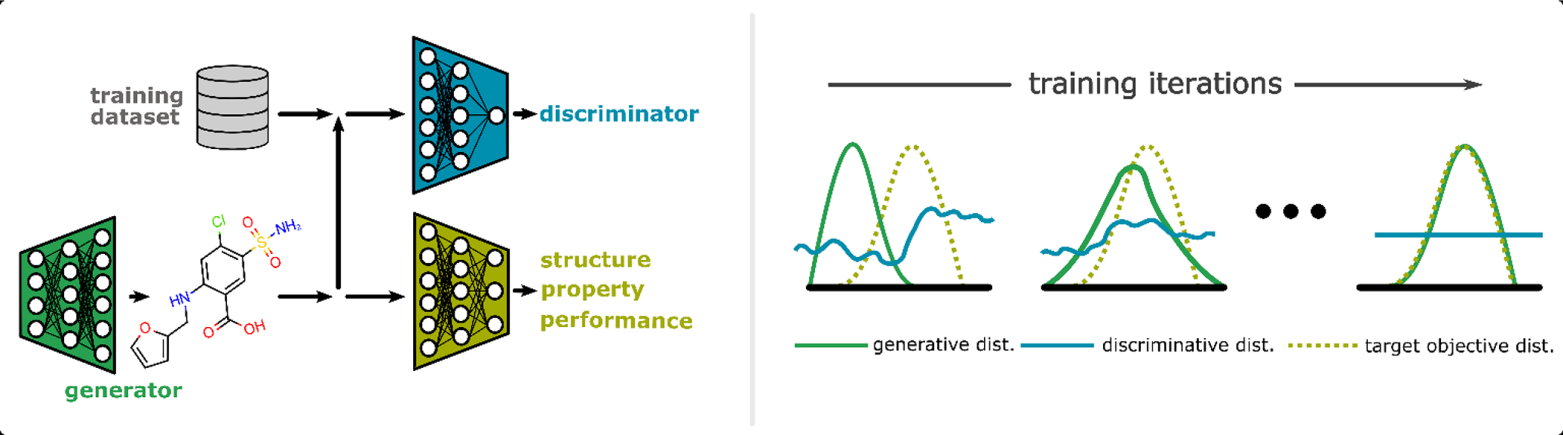
Overview of the working mechanism of an objective-guided generative adversarial network. The generator produces chemical species that are judged by the discriminator as being in- or out-of-distribution. Including an additional machine learning model for prediction biases the generation of molecules toward a desired target profile. Following several training iterations, the generative distribution resembles the target distribution, and the discriminator cannot distinguish between the two. Concepts for this figure are inspired by ref (39).

GANs can be adapted to conduct property-guided exploration by modifying the generator training with a multiobjective loss function.^41^ For example, the new loss function can be formed by weighted contributions of property predictions and the probability of mischaracterization. The generator can then simultaneously learn to optimize the target chemical characteristic alongside its usual role of deceiving the discriminator. Following multiple iterations of training, a refined set of parameters is achieved that supports generation of target-biased compounds. Training with this scheme ideally leads to a converged distribution of real chemical species that display optimal target characteristics (see Figure 2). Overall, adversarial training is a minimax problem, where the discriminator is attempting to maximize its predictive ability while the generator is attempting to minimize the generated molecule or material difference from the true distribution of species. It is important to emphasize that GAN convergence is to a saddle point that is referred to as local Nash Equilibrium. Therefore, this method is effective for generating optimal molecules in a particular region of the design space but traversing large ranges of chemistry requires further demonstration.

It is common to combine GANs with other machine learning techniques, such as RL, to build hybrid generative models.^41−44^ One of the benefits of coupling the generator with RL is that it allows for biasing the discrete molecule outputs toward a target property. For example, this was the strategy employed by You et al. in their graph convolutional policy network.^45^ They used adversarial training with proximal policy optimization, a modern RL method for policy iteration, to bias the stepwise generation of molecular graphs. One of the early uses of a hybrid property-guided model was reported by Sanchez-Lengeling et al., referred to as ORGANIC.^46^ Their model used a generator to output SMILES strings of molecules that were concurrently discriminated for the target metric and rewarded according to the REINFORCE algorithm. The MolGAN model developed by De Cao and Kipf is similar to the ORGANIC model, but instead uses a graph-based molecular input representation, an improved GAN architecture with better stability, and deep deterministic policy gradient as a RL training strategy.^47^ The more informative graph-based representation enabled better predictions on a number of benchmarking tasks.

In practice, implementing a property-guided GAN can be difficult because the effectiveness of the model is often dependent on judicious selection of hyperparameters. Readers interested in methodological instabilities of GANs are referred to refs (48)–^50^. Training is typically tuned based on accumulated trail-and-error experience of the model builder. Weighting coefficients, sample initializations, and training design can be either detrimental or enabling for the quality of exploration displayed by this class of generative models. Similar to the other methods we discuss, property-guided GANs require accurate predictions of *de novo* species to avoid exploratory failure. Future focus on developing GAN strategies is recommended, such as those that are less sensitive to parameter choices and capable of wide exploration. Immediate improvement in the average performance of GANs can be achieved by better documenting the justification for a reported model and training design. As it stands, traditional GANs maintain a status of challenging to train or are subject to failures such as mode collapse^51^—this is leading to a decrease in their popularity in favor of easier to operate methods. However, improved methodologies have emerged, e.g., Wasserstein GANs,^52,53^ that avoid common failure modes and allow for more stable training, which encourages future research into adversarial-based generation for chemical species.

### Autoencoders and Latent Spaces

It can be convenient to frame chemical discovery as an optimization problem, where the aim is to explore chemical space along an informed direction to locate performant species.^54^ Two barriers that inhibit direct optimization in chemical design spaces are worth highlighting. First, the identities of molecules and materials are discrete; in other words, a physically rational smoothly defined alchemical transformation between two species is not known. Second, to the best of current knowledge, chemical design space either is unstructured or has an immense complexity that requires high-dimensional analysis and characterization beyond the capabilities of modern chemistry. Therefore, creative methods are required to reformulate molecule or material discovery as a task amenable to direct optimization. If it is possible to convert chemical species to a continuous representation, then gradient-based optimization techniques could be applied to find critical points in the chemical design space. This forms a main motivation for using variational autoencoders (VAEs) as a generative model.^55^ A VAE is an approach to approximating a complex problem space via a lower dimensionality representation that is learned by a neural network. Successful generation of chemical species with VAEs relies on the idea of chemical encoders and decoders.

**Figure 3:**
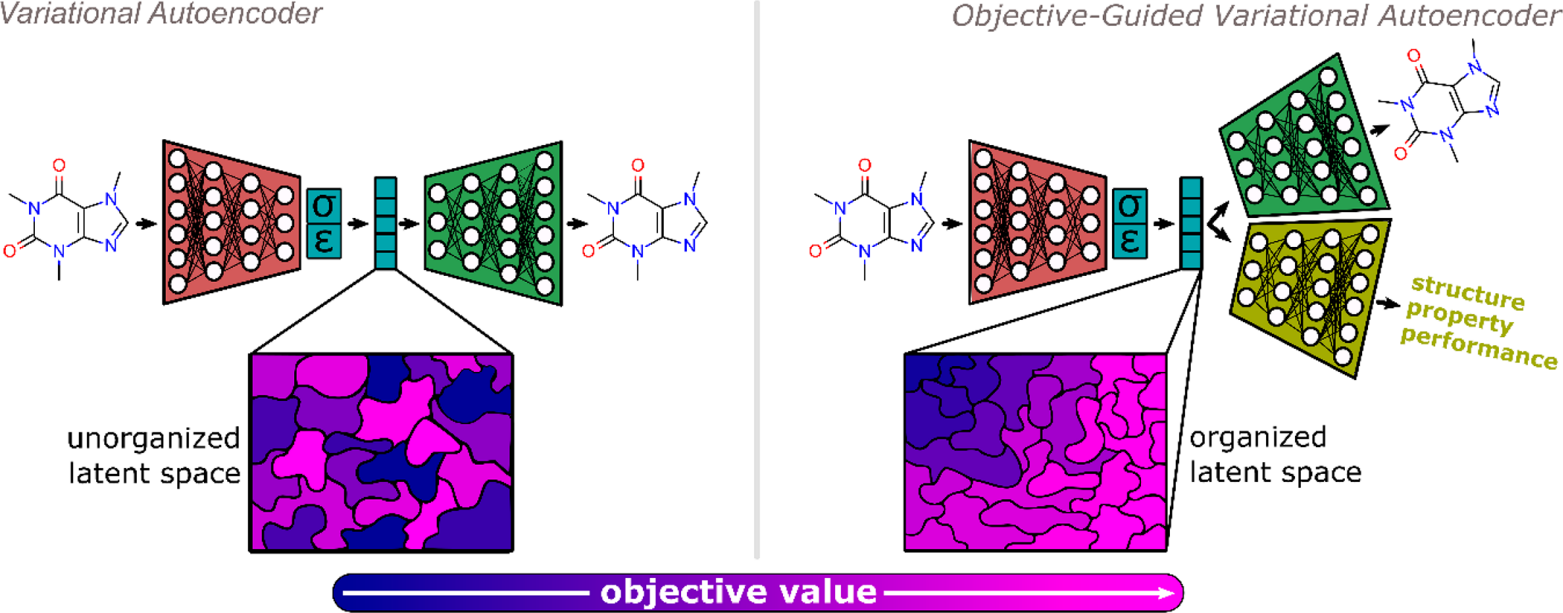
Illustration of variational autoencoder (VAE) and an objective-guided VAE for generating chemical species. VAEs operate by compressing molecules into a continuous and differentiable latent space vector. This encoder output is stochastically mapped to cover regions of the latent space using a normal distribution (mean: ε, standard deviation: σ). A reconstruction loss is used to learn the back mapping from the latent space vectors to chemical species representations via the decoder. Incorporating a machine learning model to carry out inference of the target objective organizes the latent space according to the target chemical characteristics.

The working mechanism starts with converting a molecular or material input representation into a compressed multidimensional vector. This vector corresponds to a point in a continuous and differentiable chemical space with reduced dimensionality compared to the original input, referred to as the latent space. The compression process is carried out by the chemical encoder, which is a neural network composed of a number of parameters that decrease layer-wise, i.e., a bottleneck architecture. After finding local optima in the latent space, these points need to be converted back into a human understandable chemical representation. This is the role of the decoder: a neural network that has an inverse bottleneck architecture whose final output layer matches the encoder input. During training, a VAE learns using the concept of a reconstruction loss.^56^ The objective is to minimize the difference between the chemical input representation given to the encoder and the output of the decoder. Following training, the encoder is no longer needed, and the decoder functions as a generative model for chemical discovery by converting points in the latent space into molecules or materials. The common strategy for performing property-guided species generation is to concurrently train the encoder-decoder reconstruction loss with a property prediction model operating on the latent vectors.^57^ As a result, the VAE learns to organize the latent space such that the usual decoder functionality is maintained but nearby points have similar properties (see Figure 3). The benefit of using a gradient-based optimization technique in a target-organized design space is that numerous high-performing chemical species could be generated with local sampling. Moreover, it is conceivable that a generative model operating in a chemical latent space structured according to species functionality is less likely to become trapped in a low-performance local minimum.

The first instance of utilizing a VAE as a generative model for molecular discovery was reported in the seminal work of Gómez-Bombarelli et al.^55^ Following the training of encoding and decoding SMILES strings, the authors constructed a Gaussian process model on a sampling of latent space vectors to demonstrate that molecules could be optimized directly for a target property. This finding spurred widespread interest in the generative modeling potential of VAEs. As an example, Winter et al.^58^ demonstrated that particle swarm optimization can locate optimal molecules in the latent space that outperform standard benchmarks in the GuacaMol package.^59^ Lim et al. assessed a conditional VAE for multitarget molecular discovery, where the concurrent property predictor training is replaced by coencoding and decoding of the molecular structure alongside its properties.^60^ Beyond text-based descriptors, applying VAEs to molecular graph representations is an active area of research. Simonovsky and Komodakis developed GraphVAE, which extends VAEs to operate on small graph representations while demonstrating the challenge of large molecular species.^61^ To overcome some issues of generating molecular graphs, Ma et al. introduced a regularization framework that markedly increased the validity of decoded species.^62^ Jin et al. developed a so-called junction-tree VAE that maps atom-graphs to substructure graphs and vice versa, which significantly improved the validity and property predictions.^63^ Understanding the extent that exploratory ability is reduced by operating a generative model at the level molecular substructures, i.e., graph coarse-graining, requires further study.

VAEs have become a proven tool for generative modeling tasks; however, there are a number of fundamental topics that still need to be addressed. For instance, it is necessary to develop an interpretable understanding of the implications for converting discrete entities into continuous ones. For example, the process of converting a continuous variable to a discrete one has an associated loss of details, and therefore, the reverse process introduces an arbitrary expansion of information. This operation has nonunique solutions and should be systematically studied across diverse neural network architectures, data sets, and initializations. The amount of finetuning required to achieve a functional VAE is another point. In our experience, VAEs are exceptionally data reliant, and the optimal model hyperparameters and training design is seldom transferable between tasks. Another important issue is understanding and mitigating the failures of VAEs when attempting to generate molecules or materials significantly outside the initial training data set. Incorporating physically and chemically informed constraints into the latent space organization is one conceivable approach to overcome this issue. These highlighted topics are a sampling of areas needing further investigation.

### Genetic Algorithms

**Figure 4:**
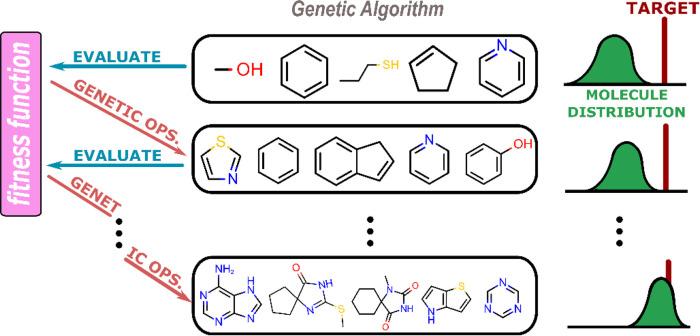
Workflow of a basic genetic algorithm for molecular discovery. The distribution of molecules composing the sample population shifts toward the target distribution through iterations of fitness evaluation and genetic operations.

Genetic algorithms belong to the larger field of evolutionary algorithms, and they are an approach to iteratively solve a discrete optimization problem.^64^ In chemistry and materials science, the optimization task commonly takes the form of maximizing a target property or fitting a defined structural feature.^65,66^ One could argue that genetic algorithms are mechanistically an optimization technique, and therefore, they are not strictly generative models. However, depending on the construction, they fulfill the functionality of discovering novel molecules or materials; thus, such a separation is moot. A basic genetic algorithm has the following steps: generation of an initial sample population, evaluate the so-called fitness of this collection of samples, mutate and crossover the most fit species to make new samples, replace unfit samples with this next generation, and repeat (see Figure 4). As a result, a generative model based on a genetic algorithm requires a molecular or material input representation that is amenable to fragment exchange modifications (crossover) and random variation (mutation). Examples of such representations include text-based strings, e.g., SMILES, fingerprints, and molecular graphs. Crossover and random mutations are the key mechanisms that enable genetic algorithms to serve as generative models. With each subsequent generation these functions allow for exploration of more fit species, i.e., those that possess the target described by the fitness function. Similar to most stochastic processes, the probability of performing various actions, the number of samples, and duration of simulation are hyperparameters that need to be tuned for efficient exploration.

Genetic algorithms have been used for decades in molecular optimization problems,^67−69^ where recent developments mainly take the form of engineering molecular representations, genetic operations, and fitness evaluation. For example, Jensen demonstrated that graph-based genetic algorithms can achieve an appreciable degree of optimization using a relatively small number of molecules and seconds of computing time.^70^ Ahn et al. reported the concept of genetic expert-guided learning that uses a deep neural network workflow to execute the genetic operations and a buffer maintaining a collection of best performing species.^71^ Kwon and Lee developed MolFinder: a variant of an evolutionary algorithm that carries out optimization using a distance-based constraints on molecular similarity that decays with each new generation.^72^ Maintaining a minimum similarity distance is motivated by the desire to avoid having too many molecules sampled from the same local chemical space. More recently, Nigam introduced an approach called JANUS, which maintains two separate populations with genetic operations tailored for explorative and exploitative purposes.^73^ Their methodology is adaptive and performs well on many inverse design benchmarks. These examples highlight that genetic algorithms have a proven utility for generating small organic molecules, especially when they are combined with other data-driven techniques.^74^

It is possible for genetic algorithms to exceed the performance of machine learning approaches as generative models,^75^ especially when the scientific discovery task is bound by a collection of well-defined building blocks. For instance, one appropriate use of a genetic algorithm could be to generate sequenced define polymers with a particular property from a collection of known monomer chemistries.^76^ It is worthwhile to highlight two deficiencies of genetic algorithm generative models. The first is the propensity to converge to local optima.^77^ This issue can be mitigated to an extent by choosing a well-diversified initial population; however, its avoidance requires the inclusion of a mechanism to sacrifice immediate fitness for the progress of future generations of molecules and materials. It also possible to simply restart the process after fitness is no longer improving for many generations.^78^ The second deficiency is the comparable functionality to Edisonian-based research. In the introduction we pointed out the inefficiency of trial-and-error approaches, and a similar critique can be applied to genetic algorithms. Assuming premature convergence is not an issue, finding creative molecules and materials that are significantly different than structures composing early sample generations could require the evaluation of a prohibitively large number of iterations. These points should not distract from the fact that a well-designed genetic algorithm and properly constructed chemical population can outperform modern and complex generative approaches; for example, see CReM.^79^

### Generative Flows and Diffusion

**Figure 5:**
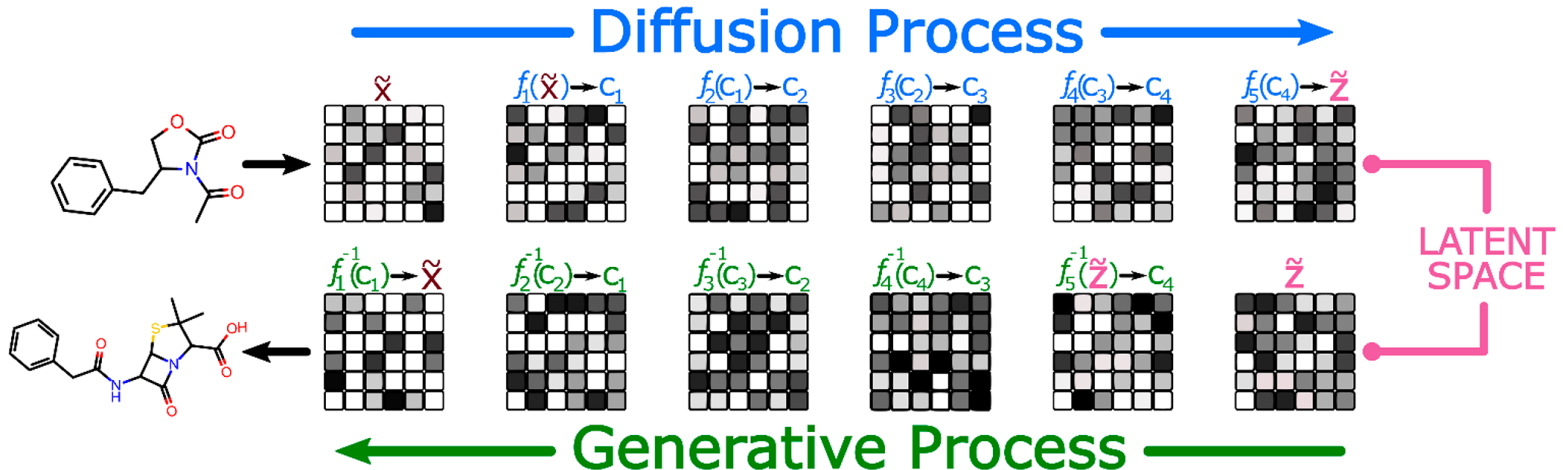
Overview of using diffusion-based methods to generate chemical species. The generative process consists of applying a series of gradual denoising steps to a sampled latent space vector that ultimately maps to a chemical species representation.

Those interested in generative modeling research outside of the chemical sciences are likely familiar with fascinating models such as stable diffusion, where a text-based description can be converted to a corresponding image.^80^ These models are based on the concepts of normalizing flows and diffusion, and it is worth considering if such methods are useful for generative modeling in the chemical sciences.^81^ Similar to VAEs, the working mechanism of normalizing flows and diffusion models for chemical discovery is to produce species by sampling latent representations. From a high-level view, the generative process occurs by learning a number of sequential steps that gradually transform a latent vector to a chemical representation. In the normalizing flow approach, models learn to convert chemical representations into latent space vectors and vice versa using invertible functions. Diffusion-based models are similar to normalizing flows with the exception that the forward and inverse deterministic functions are replaced with stochastic operations, which effectively learn to add and subtract random noise (see Figure 5). There is resemblance between these methods and the previously discussed VAEs because both generative mechanisms rely on learning to convert latent space vectors. Similar unanswered research questions are relevant, for example, the implications of treating discrete chemical compounds with continuous representations. Overall, normalizing flows and diffusion-based generative models are an interesting yet minimally explored area, where innovation could result in the unique discovery of de novo chemistry.

Flow-based models are one of the newest generative strategies computational chemists are exploring, and thus, only a handful of reports using these techniques have been made. One of the most notable works is the GraphAF model by Shi et al., where the authors implemented an autoregressive end-to-end molecular graph generator using normalizing flows.^82^ For a similar purpose as other previously highlighted models, the authors implement RL, specifically, proximal policy optimization, alongside their autoregressive flow model to bias their graph generation toward specific chemical targets. Overall, GraphAF was shown to outperform other common generative models at the time in its ability to generate high penalized logP values. While these benchmarks are arbitrary in their direct scientific value, the reported performance supports that normalizing flows should be considered among the major generative modeling strategies. While we have focused on high-level details, it is worth highlighting that the mathematics and underlying implementation are more involved than the previously discussed strategies. There have been a small number of additional reports that apply flow-based concepts to generate small organic molecules; for example, see Kutznetsov and Polykovskiy,^83^ Satorras et al.,^84^ Madhawa et al.,^85^ and Hoogeboom et al.^86^ In summary, normalizing flows and diffusion have joined the growing toolkit of computational chemists interested in generative modeling, and their potential is largely untested.

## Going Forward with Generative Models

### Methodology and Metrics

#### Adopting Standard Reporting Practices

Machine learning-based generative models require accurate data sets that can be readily accessed. The value of curating high-quality data sets is important; for instance, those that are well-characterized, presented in common formats, and require minimal processing steps in preparation for training can accelerate the development of new generative strategies. From the perspective of dissemination, a detailed understanding of the initial data set is essential for interpreting the quality of results and the novelty of generative model innovations. Standard data reporting practices should be agreed upon and adopted for the continued development of generative models; for example, see Wilkinson et al.^87^ In addition to the data sets, thorough reporting practices for generative models should be followed. From our experience, it is common for a range of small to important implementation details to be left out of chemistry focused articles. We anticipate that this could lead to compounding issues of reproducibility, difficulties for those looking to join the generative modeling field, and an unnecessary slowing of progress. Therefore, it is encouraged to deposit complete scripts, code (with useful comments), parametrizations, trained model parameters, and well-written documentation into public repositories when presenting a new generative model.^88^ One motivation could be to adopt a similar mindset to so-called TRUE molecular simulations: transparent, reproducible, usable by others, and extensible.^89^

#### Quantitative Measures of Generative Performance

It is straightforward to informally state the desired performance criteria for a generative model: for example, “does my model produce realistic, diverse, and high-performing molecules outside the distribution of known species?” However, performing meaningful quantitative analysis that reflects the answer to such a question is more complicated. A number of challenges related to this point are discussed by Renz et al.^90^ Considering that inverse design via generative models is an emerging paradigm, there is no precedent for the best metrics to describe different generative models. The statistics that are appropriate for a given generative model depend on the mechanism employed and the input and output representations. Those interested in an extended discussion on molecular representations are directed to the work of David et al.^91^ Text-based molecular representations (e.g., SMILES or SELFIES^92^) could benefit from quantitative measures that are applied in the field of natural language processing, such as perplexity and cross-entropy. Unfortunately, these measures do not possess any directly interpretable value for solving chemical science challenges. Quantitative measures for different generative methods are an active point of interest in the computer science community, and several mechanism specific reports have been made: Borji for GAN,^93,94^ Chen et al. for latent space methods,^95^ and Henderson et al. for RL.^96^ It is possible that research efforts in these directions will lead to useful metrics irrespective of the application; however, pursuing chemistry-specific alternatives appears to be a more likely solution. In summary, it is critical that studies focused on generative mechanisms apply targeted quantitative measures that describe the performance for a scientific application. Moreover, we also to need address methodological questions such as the likelihood a model will generate a diverse species, the amount of deviation from the known data set, and the extrapolation-exploitation trade-off. These points do not alone deem one model to have greater scientific value than another, but they are indicators of the overall development of generative modeling practices in the chemical sciences.

The process of comparing different models on standard data sets and tasks, i.e., benchmarking, is an important aspect of the development process. This allows for insight into the underlying mechanisms that lead to better performance in a target area. As an example, Gao et al. presented a thorough benchmarking study for molecular optimization that emphasized the importance of sample efficiency, the effects of input representations, and the suitability of various methods for diverse tasks.^75^ However, comparison between generative modeling strategies is challenging to carry out because of the diversity of strategies used for exploration/exploitation and discovery. Polykovskiy et al. outline several metrics in the MOSES benchmarking platform that can be used to evaluate generative model performance.^97^ Similarly, Brown et al. have created an evaluation framework called GuacoMol, which offers a collection of standardized generative modeling benchmarks.^59^ We encourage further development of strategies to accurately compare generative models on a diversity of tasks. Multiobjective analysis, such as the previously mentioned synthesizability–performance combined loss, can result in a Pareto front-like collection of solutions, and related appropriate benchmarks are currently lacking.^98^ An equally needed benchmarking task for existing and future generative models is the analysis of computational scalability metrics. As a final point, we advocate for meaningful benchmarking, but the value of such a practice should be cautiously interpreted. A definitive claim to outperforming another model on a particular benchmarking task does not necessarily equate to greater scientific value. Equitable benchmarking of generative models is, in our opinion, an ambiguous task. Demonstrations of one method uniquely achieving discovery over others in an application or experimental setting is an appealing future research direction.

#### Scalability and Efficiency

The availability of tutorials and software toolkits make it possible for anyone with reasonable determination to perform machine learning tasks; however, this can lead to sacrificing efficiency and proper programming practices. Our experience with the open-source repositories associated with molecule or material generative model publications is that a trend exists of code written to favor pragmatism over efficiency. While this does not hinder short-term scientific reports, long-term generative model progress will assuredly have a component of models trained on big data parallelizable over large heterogeneous computing resources. One step toward realizing such models is to invest in educating chemical science researchers on strategies for maximizing machine learning model efficiency on resources provided by supercomputing centers. Oftentimes the key to achieving excellent machine learning models is in the programming details: these range from subtle topics like data precision to more complex design decisions such as model and data parallelization strategies. An area to draw inspiration for the importance of efficiency is natural language processing, which, in recent years, has used both *big data* and *big models* to achieve exceptional results.^99,100^ Generative models for molecule and material discovery tasks should look to embrace a similar scale for complex applications. In our experience, generative models typically become useful with tens to hundreds of thousands of samples, where fine-tuning the utility and exploiting discovery capabilities often requires several orders of magnitude more data.

#### Interpretability

The step beyond generating a high-performance molecule or material is to understand the mechanism by which a model arrived at the target.^101,102^ Explainable generative models with an interpretable basis can refine chemical understanding. A difficulty with analyzing generative mechanisms is that data-driven techniques can effectively operate in high-dimensional spaces, which poses a challenge for human researchers. A frequented tool for general interpretability analysis is the use of dimensionality reduction techniques, such as t-SNE^103^ or UMAP,^104^ that allow high-dimensional data to be visualized with a low-dimensional representation. Generative model specific analysis can also be performed depending on the mechanism used, where different models vary in ease of interpretability. Genetic algorithms are among the most interpretable models because persistent substructure and chemical motif analysis can be performed across several generations of improving fitness. There have also been creative visualization procedures reported for understanding the exploratory behavior of genetic algorithms; for example, see Leguy et al.^105^ Other generative modeling approaches like VAEs have mainly relied on techniques such as t-SNE and UMAP for interpretability. To understand the exploratory ability of these methods, it is possible to carry out distance-based analysis in the latent space, however, this should be viewed as a qualitative measure because the distance between two points is nonunique and depends on model initialization/training. RL is arguably the least interpretable method we have discussed because of the large amount of analysis needed to determine the state-action space a model is learning along a sequence of experiences. Pursuing the development of interpretability techniques and insightful visualizations that shed light on the chemically relevant inner workings of a generative model are areas in need of focus.

#### Embracing Active Learning

Active learning refers to the practice of including considerations for processing new data in training or inference. As an example, active learning can be used during training to limit the inclusion of data points to those that improve that target function of a model. Query-by-committee is one popular strategy, where uncertainty is calculated by the disagreement between multiple models that are trained on the same data set with different initializations.^106^ The interested reader is directed to Smith et al. for a demonstration of query-by-committee being used to reduce training data set size without sacrificing accuracy.^107^ Most generative modeling strategies can benefit from the use of active learning as part of their workflow. For instance, active learning can be used to check the accuracy of machine learning-based fitness functions in genetic algorithms, the reward functions in RL, property biases in GANs, and the uncertainty of the decoder in VAEs. It is also possible to integrate active learning directly into the sample generation process, for example, see the work of Zhu and Bento for generative adversarial active learning.^108^ Active learning is particularly important for property-guided generative models with large exploratory abilities. Mischaracterizing the target properties of a *de novo* molecule or material that is significantly outside the known distribution can be detrimental to the discovery process, and active learning is one approach to combat this. Fortunately, the continuous increase in computing capabilities is reducing the burden of active learning tasks, for example, parallel training of multiple models to perform query-by-committee. Adopting efficient training and target uncertainty reporting via active learning is an encouraged future standard.

### Applied Generative Modeling

#### Connecting with Synthetic Chemistry

A clear interest exists in using generative models to automate chemical discovery tasks; however, the usefulness of this strategy is restricted depending on the synthetic feasibility of the suggested molecule or material building block.^109^ Even if a synthetic pathway exists for a generated *de novo* species, it must be amenable to the scale-up restrictions for a target application. More directly, species that have low yields and numerous synthetic steps, exotic chemistries, and/or harsh reaction conditions are unlikely to have widespread meaningful impact. It is worthwhile to adopt a standard practice of postprocessing generative models with reaction network characterization, retrosynthetic analysis, or empirical synthesizability scores as a short-term strategy. However, we anticipate that the long-term utility of generative models will be best realized through an intimate connection with the constraints of synthetic chemistry. Recent progress in this direction includes employing RL to build synthetic trees using reaction templates^110^ and a conditional VAE that jointly encodes molecules with synthetic reaction sequences, to name a few.^111^ Future generative models should have mechanisms for biasing discoveries toward creative and performant species that can be readily synthesized. Several ideas are being explored such as Monte Carlo tree search,^112,113^ directed acyclic graph analysis,^114^ and template-free transformers,^115^ to name a few. It might be possible to bias generation of feasible chemical species by engineering a loss function that includes a contribution for synthesizability. However, a few points need to be considered. *De novo* molecules might be a result of *de novo* synthetic chemistry, and thus, implementing a loss function with synthesizability constraints requires a system that is dynamic enough to identify such pathways. Furthermore, the process of exploration and exploitation becomes more complicated. For example, generative models operating with synthesizability constraints may need to balance the value of sacrificing immediate target performance to locate a more synthesizable molecule. We anticipate that systematic analysis in this area will be critically important.

#### Connecting with Experimentation

Ongoing efforts exist to build automated experimentation platforms, where the end goal is a system that operates with minimal human intervention to discover chemical compounds given enough time, resources, and functionality. For an automated discovery platform to act as a self-driving system it must be able to identify novel chemical species and plan a synthetic strategy. This is an emerging field of research; however, the reported successes are promising.^20,116,117^ To enable generative models to directly contribute to solving challenges in the chemical sciences, there is value in connecting them with automated experimentation. It is interesting to consider incorporating practical constraints into a generative model that operates as a selector in a self-driving discovery task. As an example, future autonomous laboratories will likely have access to a fixed number of reactants and supported synthesis conditions. Therefore, it is worthwhile to explore biasing molecule or material generation to answer questions such as “what is the highest performing chemical species that can be generated within the tolerances of this robotic platform?” The short-term answer to restricted discovery could be an engineered multiobjective loss function. However, we expect that this approach will not have longevity as the complexity of the discovery task increases over time, and a collaborative effort between generative modelers and automation experimentalists will be needed for a robust solution. Unfortunately, the accessibility of automated experimentation is limited. The development of accessible, small-scale, and cost-effective testing platforms that can be used by synthetic/computational chemists to develop practical generative models could be of interest.

#### Macromolecules, Materials, and Assemblies

Macromolecules and materials with large building blocks are challenging species to apply generative models to. On one hand, the size of these species introduces difficulties for scalable and informative molecular representations. Macromolecular input representations are emerging, such as BigSMILES,^118^ but they have yet to be applied in a generative modeling framework. One of the common strategies to avoid descriptors of large macromolecules and assemblies is to assume that polymer-level properties can be inferred from details of the building block chemistry, i.e., end-to-end predictions. This has shown to be successful for discriminative models; for example, see the polymer genome.^119^ The area of applying generative models to polymeric systems could be enhanced by the development and application of creative large-molecule representations. On another hand, material informatics techniques are limited because of the amorphous structure that many polymeric materials display. This has led to a scarcity of training data for such systems, and as a result, generative models for amorphous structures have been minimally explored. To support the development of generative models for amorphous systems, high-throughput methods should be pursued that enable rapid atomistic model building and measurement. Efforts in this area are key to curate data sets with sufficient details and chemical coverage for accelerated discovery of macromolecular species. Regarding ordered structures, such as organic crystals, methods that can generate unit cells and molecular packing can have wide reaching impact for this important class of functional materials; for example, see the recent work of Köhler et al.^120^

### Generative Modeling *in Silico*

#### Engineering Discovery as Reinforcement Learning

We have described that RL techniques are defined by their treatment of states, actions, and rewards. These three components need to be engineered to fit the discovery task for utilizing RL in a generative modeling framework. In particular, choosing a reward functional form is critically important for the explorative and exploitative ability of a generative model. We are currently unaware of studies that systematically vary a multiobjective reward function and report effects on the generative model performance; however, there is significant perceived value in such investigations. RL approaches maintain a position of difficult to train without prior trial-and-error experience, and therefore, the development of robust modeling methodologies is an ongoing need. Training strategies can be broadly classified as online, off-policy, and offline RL. From the standpoint of robustness, offline RL is the most attractive among these three. It is worth emphasizing that our comments on offline RL-based generative models are motivated by simpler training and are not an endorsement of their performance over online or off-policy strategies. The offline RL paradigm is defined by learning the state-action space via rewards using an established data set of experiences. This setup provides a RL solution that is less sensitive to hyperparameters because the data set is fixed. However, two common issues resurface: (1) the data sets for training need to be curated and made available, and (2) the model’s initial ability to fit the state-action space is dependent on the composition of the data set. Further development of offline RL for generative modeling is needed.

#### Integration with Machine Learned Potentials

The major generative methods highlighted in this perspective all have an important commonality; namely, they rely on efficient and accurate evaluation of the target. Many target properties are derivative of the potential and free energies of interaction, and thus, their rapid analysis can enable generative modeling success. Among the most accurate approaches for characterizing interaction energies and forces are quantum mechanical calculations, where one notable example is density functional theory. However, these calculations are too time-consuming to integrate into the large data volume workflow stages of a generative model, and therefore, they are mainly used for testing when applicable. This is especially true for large molecular or material systems such as polymers.^121^ An attractive alternative is to replace quantum mechanical calculations with machine learned interatomic potentials (MLIPs).^122^ If carefully constructed, these methods can achieve near-density functional theory accuracy with several orders of magnitude reduced computational cost, which makes them conceivably useful components of generative models. The MLIP field is rapidly expanding and growing in sophistication: a number of accurate machine learned potentials have been reported, and recent efforts are including long-range interactions.^123^ For a recent example of MLIP utility, the work of Rufa et al.^124^ uses ANI2x^125^ to improve the accuracy of absolute binding free energy calculations to 0.5 kcal/mol for protein–ligand systems, thus providing accuracy that could be used by generative models aimed at small molecule drug discovery. We envision concerted efforts for developing MLIPs alongside generative modeling strategies will be key to future discovery.

#### Innovative Generative Strategies

Progress in generative models for chemistry has been continuous, but effective implementation in a closed-loop discovery system is still in its infancy. Many generative models maintain a position of difficult to implement, train, and apply. Development of robust strategies capable of efficient target-guided exploration or extrapolation is an area in need of focus. It is worth pointing out that a significant number of reports on generative models could be classified as being adapted from general methodological research occurring in the field of computer science. It is unclear if the development of generative models in the chemical sciences is hindered or bolstered by this adaptation. There could be value in pursuing the development of generative strategies that are specific to search, discovery, and refinement challenges faced by the chemical sciences. As an example, it is worth questioning whether general RL algorithms that can solve problems such as “cart-pole” are best suited for finding molecules or materials. Similar statements can be made for the other generative methods discussed.

#### Other Generative Modeling Opportunities

For the majority of this perspective, we have focused on discovery as the process of suggesting chemical compositions or structures via guided generative models. Generative models can also support molecule or material discovery through performing useful tasks outside of *de novo* species identification. Focusing on the field of materials discovery, it can be necessary to construct atomistic or coarse-grained models to obtain a necessary data set for inferring properties from microstructural features. Generative models have a conceivable ability to efficiently accomplish standard modeling tasks at longer length scales that would otherwise need to be performed manually, such as the partitioning of collections of atoms into coarse-grained beads.^126,127^ In the field of crystal structure predictions, a generative model could serve as the mechanism for finding the optimal packing geometry at target thermodynamic conditions. For single molecule screening, it can be beneficial to leverage features from the 3D molecular structure. Producing low-energy 3D conformer geometries from text-based molecular input representations is an area that can benefit from generative model development. TorsionNet is one example of progress toward accomplishing such a task, where RL was used to develop a conformer sampling strategy that operates on rotatable bonds.^128^ These instances are emblematic of the diverse opportunities available for generative modeling developments. Creative formulation and implementation of generative models across different steps of *in silico* design and experimental discovery processes are worth further investigating.

## Concluding Remarks

Applied generative modeling offers an opportunity to reformulate design and discovery in the chemical sciences. Examples of generative models that suggest unexplored chemical species are now abundant. We believe it is time to look beyond demonstrating the operability of generative models and move toward their practical implementation for solving scientific challenges. Arguably, a *holy grail* of modern chemical research is the implementation of an efficient closed-loop discovery process, where target molecules or materials are generated, synthesized, characterized, and refined with minimal human input. This can be in the form of self-driving experimentation or autonomous quantum mechanical calculations followed by synthetic chemistry, to name a few. Considering the impact that generative modeling is expected to have on the future of chemical research, collaboration between computational chemists and synthetic chemists is a necessity.

This perspective has highlighted that many unique strategies exist outside of human intuition for exploring interesting molecules in chemical design spaces. It is not possible to predict the exact nature of future chemistry and materials research; however, the established success of generative modeling approaches up to this point indicates that these techniques will continue to be present. It is our opinion that certain chemical research practices, such as molecular discovery by combinatorics, are best handled by machines, and this is a point that should be willingly accepted. This is not to say that we are envisioning that generative models will drive the obsolescence of human scientists. On the contrary, a successful generative model can be an exceptional tool for discovering novel molecules and materials that may otherwise be unthinkable using current chemistry, thus, pushing human scientists to reconsider and improve their chemical understandings. Even in failure, generative models can construct diverse *in silico* chemical libraries that may inspire creative innovation among chemists and materials scientists. Application-focused generative modeling will be a key step in accelerating chemical research to the point where implementable solutions to pressing challenges can be realized. Generative models are at the core of an emerging style of research, where the human chemist points in a direction and constructs a machine to formulate a hypothesis and investigate it.
